# (*E*)-1-(3-Nitro­phen­yl)-2-({5-[(1*E*)-2-(3-nitro­phen­yl)hydrazin-1-ylidenemeth­yl]-2-thien­yl}methyl­idene)hydrazine

**DOI:** 10.1107/S1600536810002771

**Published:** 2010-01-27

**Authors:** Geraldo M. de Lima, William T. A. Harrison, Edward R. T. Tiekink, James L. Wardell, Solange M. S. V. Wardell

**Affiliations:** a Departamento de Quimica, ICEx, Universidade Federal de Minas Gerais, 31270-901 Belo Horizonte, MG, Brazil; b Department of Chemistry, University of Aberdeen, Meston Walk, Old Aberdeen AB24 3UE, Scotland; c Department of Chemistry, University of Malaya, 50603 Kuala Lumpur, Malaysia; d Centro de Desenvolvimento Tecnológico em Saúde (CDTS), Fundação Oswaldo Cruz (FIOCRUZ), Casa Amarela, Campus de Manguinhos, Av. Brasil 4365, 21040-900 Rio de Janeiro, RJ, Brazil; e CHEMSOL, 1 Harcourt Road, Aberdeen AB15 5NY, Scotland

## Abstract

The title mol­ecule, C_18_H_14_N_6_O_4_S, adopts a U-shape with the aromatic groups lying *syn* and oriented in the same direction as the thio­phene S atom. Twists away from planarity are evident with the maximum deviation being found for a terminal nitro group: C/C/N/O = 19.0 (3)°. The conformation about each of the C=N bonds is *E*. In the crystal, centrosymmetrically related mol­ecules are connected *via* N—H⋯O_nitro_ hydrogen bonds, forming 14-membered {⋯HNC_3_NO}_2_ synthons. These are linked into layers *via* C—H⋯O_nitro_ inter­actions with the primary inter­actions between layers being of the type C—H⋯π, where the π-system is the thio­phene ring.

## Related literature

For the preparation of hydrazones of thio­phene­carbaldehydes, see: Kwon *et al.* (2009 ▶); Wardell *et al.* (2007 ▶); Vaysse & Pastour (1964 ▶). For general uses of 2-substituted-thio­phenes, see: Campaigne (1984 ▶). For their specific uses as materials, see: Michaleviciute *et al.* (2007 ▶, 2009 ▶); Kwon *et al.* (2009 ▶). For their specific uses as pharmacological agents, see: Kleemann *et al.* (2006 ▶); Sonar & Crooks (2009 ▶); Mellado *et al.* (2009 ▶); Satyanarayana *et al.* (2008 ▶); Lourenço *et al.* (2007 ▶). For related structures, see: Wardell *et al.* (2007 ▶, 2010 ▶); Ferreira *et al.* (2009 ▶); Nogueira *et al.* (2010 ▶).
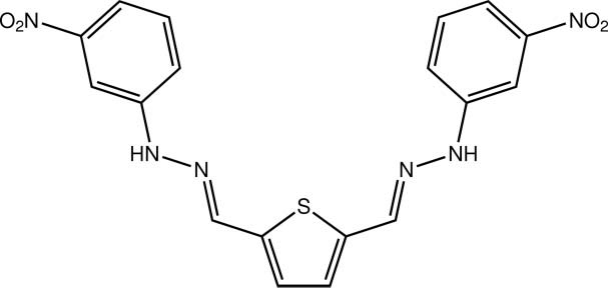


## Experimental

### 

#### Crystal data

C_18_H_14_N_6_O_4_S
*M* *_r_* = 410.41
Monoclinic, 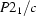

*a* = 11.1790 (5) Å
*b* = 20.6993 (9) Å
*c* = 8.0334 (2) Å
β = 100.513 (2)°
*V* = 1827.70 (12) Å^3^
*Z* = 4
Mo *K*α radiation
μ = 0.22 mm^−1^
*T* = 120 K
0.62 × 0.10 × 0.06 mm

#### Data collection

Nonius KappaCCD area-detector diffractometer
Absorption correction: multi-scan (*SADABS*; Sheldrick, 2007 ▶) *T*_min_ = 0.668, *T*_max_ = 0.746
21780 measured reflections
4183 independent reflections
3001 reflections with *I* > 2σ(*I*)
*R*_int_ = 0.071

#### Refinement

*R*[*F* ^2^ > 2σ(*F* ^2^)] = 0.046
*wR*(*F* ^2^) = 0.133
*S* = 1.08
4183 reflections
268 parameters
H atoms treated by a mixture of independent and constrained refinement
Δρ_max_ = 0.27 e Å^−3^
Δρ_min_ = −0.34 e Å^−3^

### 

Data collection: *COLLECT* (Hooft, 1998 ▶); cell refinement: *DENZO* (Otwinowski & Minor, 1997 ▶) and *COLLECT*; data reduction: *DENZO* and *COLLECT*; program(s) used to solve structure: *SHELXS97* (Sheldrick, 2008 ▶); program(s) used to refine structure: *SHELXL97* (Sheldrick, 2008 ▶); molecular graphics: *ORTEP-3* (Farrugia, 1997 ▶) and *DIAMOND* (Brandenburg, 2006 ▶); software used to prepare material for publication: *publCIF* (Westrip, 2010 ▶).

## Supplementary Material

Crystal structure: contains datablocks global, I. DOI: 10.1107/S1600536810002771/hg2635sup1.cif

Structure factors: contains datablocks I. DOI: 10.1107/S1600536810002771/hg2635Isup2.hkl

Additional supplementary materials:  crystallographic information; 3D view; checkCIF report

## Figures and Tables

**Table 1:** Hydrogen-bond geometry (Å, °)

*D*—H⋯*A*	*D*—H	H⋯*A*	*D*⋯*A*	*D*—H⋯*A*
N2—H2n⋯O2^i^	0.89 (2)	2.27 (2)	3.103 (2)	156 (2)
C2—H2⋯O3^ii^	0.95	2.46	3.278 (3)	145
C18—H18⋯O4^iii^	0.95	2.48	3.241 (3)	137
C12—H12⋯*Cg*1^iv^	0.95	2.58	3.323 (2)	135

Symmetry codes: (i) 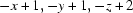; (ii) 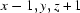; (iii) 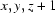; (iv) 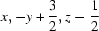.

**Fig. 1.**
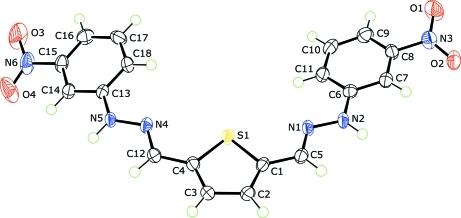
The molecular structure of (I) showing the atom-labelling scheme and displacement ellipsoids at the 70% probability level.

**Fig. 2.**
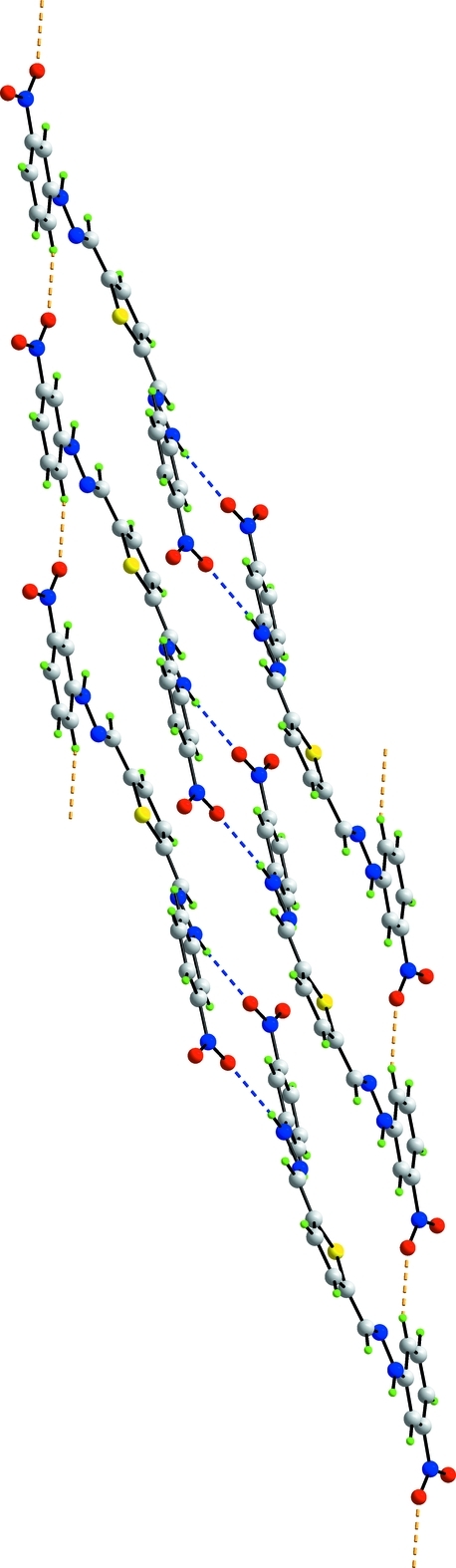
A view of the supramolecular chain in (I) whereby centrosymmetrically related dimeric aggregates, held together by N–H···Onitro hydrogen bonds (blue dashed bonds), are linked via C–H···Onitro interactions (orange dashed lines). Colour code: S, yellow; O, red; N, blue; C, grey; and H, green.

**Fig. 3.**
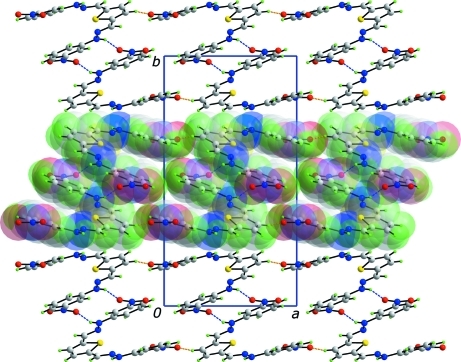
View of the stacking of layers in (I) with the central layer highlighted by a superimposed space filling representation. The N–H···O and C–H···O interactions are shown as blue and orange dashed lines, respectively. Colour code: S, yellow; O, red; N, blue; C, grey; and H, green.

## supplementary crystallographic information

### Comment

The preparation of hydrazonederivatives of thiophenecarbaldehydes is well documented (Kwon, *et al.* 2009; Wardell *et al.*, 2007; Vaysse & Pastour, 1964). As a continuation of structural studies of this class of compound (Wardell *et al.*, 2007; Ferreira *et al.*, 2009; Nogueira *et al.*, 2010; Wardell *et al.*, 2010), the title compound (I) was synthesised and structurally investigated. 2-Substituted thiophenes in general have various uses, for example as dyestuffs, flavour agents, drugs, and inhibitors (Campaigne, 1984). Thiophenes are present in many natural and synthetic products with a wide range of pharmacological activities (Kleemann *et al.*, 2006; Sonar & Crooks, 2009; Mellado *et al.*, 2009; Satyanarayana *et al.*, 2008; Lourenço *et al.*, 2007). Specifically, hydrazone derivatives of thiophene have found uses in optoelectronic applications (Michaleviciute *et al.*, 2007), as optical non-linear materials (Kwon *et al.*, 2009), and as hole transporting materials (Michaleviciute *et al.*, 2009).

The overall molecular conformation in (I) is U-shaped as the two aromatic residues lie to the same side of the molecule and are syn, being orientated in the same direction as the thiophene-S atom, Fig. 1. Twists from planarity in the molecule are evident in each of the side-arms, i.e. about the N2–C6 and N4–N5 bonds; the N1/N2/C6/C7 and C12/N4/N5/C13 torsion angles are 168.57 (18) and -170.79 (19) °, respectively. In addition, the values of the C7/C8/N3/O1 and C14/C14/N6/O3 torsion angles of -161.0 (2) and 164.4 (2) °, respectively, indicate each of the nitro groups is twisted out of the plane of the benzene ring to which it is bonded. The conformation about each of the C5═N1 [1.288 (3) Å] and C12═N4 [1.285 (3) Å] double bonds is *E*.

In the crystal packing, centrosymmetrically related molecules associate via *N*–*H*···O_nitro_ hydrogen bonds to result in the formation of a 14-membered {···HNC_3_NO}_2_ synthon, Table 1. The dimeric aggregates are linked into a supramolecular chain along the *c* axis via *C*–*H*···O_nitro_ interactions, Table 1 and Fig. 2. The chains in turn are linked into layers in the *ac* plane via further *C*–*H*···O_nitro_ interactions, Table 1. The layers thus formed stack along the *b* axis with the primary interactions between them being of the type C–H···π where the π-system is the thiophene ring [C12–H···ring centroid(S1,C1–C4)^i^ = 2.58 Å, C12···ring centroid^i^ = 3.323 (2) Å with an angle subtended at H = 135 ° for symmetry operation *i*: *x*, 3/2-*y*, -1/2+*z*]. The second N5-amine H does not participate in a formal hydrogen bond. It is noted that the N4–N5—H residues lie in the interlayer region and are in relative close proximity [e.g. N5–H···N4^ii^ = 2.87 Å] but steric constraints preclude a closer approach of these groups to allow a hydrogen bonding interaction.

### Experimental

Solutions of 3-nitrophenylhydrazine.hydrochloride (0.38 g, 2 mmol) in EtOH (10 ml) and 2,5-thiophenedicarbaldehyde (0.14 g, 1 mmol) in EtOH (10 ml) were mixed. The reaction mixture was refluxed for 1 h, and rotary evaporated. The solid residue was recrystallised twice from aq. EtOH (v:v 1:2), m.p. 497-479 K. ^1^H NMR (400 MHz, DMSO-d_6_): δ 7.26 (s,2*H*), 7.41 (dd, 2H, J = 1 & 8.1 Hz), 7.51 (t,2*H*, J = 8.0 Hz), 7.59 (dd, 2H, J = 1.3 & 8.0 Hz), 7.79 (t, 2H, 2.0 Hz), 8.10 (s, 2H), 10.91(s, 2H) p.p.m. ^13^C (100 MHz, DMSO-d_6_): δ 105.7, 113.10, 118.2, 128.5, 130.5, 134.3, 140.1, 145.9, 148.8 p.p.m.

### Refinement

The C-bound H atoms were geometrically placed (C–H = 0.95 Å) and refined as riding with U_iso_(H) = 1.2U_eq_(C). The N-bound H atoms were located from a difference map and refined with U_iso_(H) = 1.5U_eq_(N).

### Crystal data

**Table:**

C_18_H_14_N_6_O_4_S	*F*(000) = 848
*M**_r_* = 410.41	*D*_x_ = 1.492 Mg m^−^^3^
Monoclinic, *P*2_1_/*c*	Mo *K*α radiation, λ = 0.71073 Å
Hall symbol: -P 2ybc	Cell parameters from 13699 reflections
*a* = 11.1790 (5) Å	θ = 2.9–27.5°
*b* = 20.6993 (9) Å	µ = 0.22 mm^−^^1^
*c* = 8.0334 (2) Å	*T* = 120 K
β = 100.513 (2)°	Rod, red
*V* = 1827.70 (12) Å^3^	0.62 × 0.10 × 0.06 mm
*Z* = 4	

### Data collection

**Table:**

KappaCCD area-detector diffractometer	4183 independent reflections
Radiation source: Enraf Nonius FR591 rotating anode	3001 reflections with *I* > 2σ(*I*)
10 cm confocal mirrors	*R*_int_ = 0.071
Detector resolution: 9.091 pixels mm^-1^	θ_max_ = 27.5°, θ_min_ = 3.1°
φ and ω scans	*h* = −14→14
Absorption correction: multi-scan (*SADABS*; Sheldrick, 2007)	*k* = −26→26
*T*_min_ = 0.668, *T*_max_ = 0.746	*l* = −10→9
21780 measured reflections	

### Refinement

**Table:**

Refinement on *F*^2^	Primary atom site location: structure-invariant direct methods
Least-squares matrix: full	Secondary atom site location: difference Fourier map
*R*[*F*^2^ > 2σ(*F*^2^)] = 0.046	Hydrogen site location: inferred from neighbouring sites
*wR*(*F*^2^) = 0.133	H atoms treated by a mixture of independent and constrained refinement
*S* = 1.08	*w* = 1/[σ^2^(*F*_o_^2^) + (0.0678*P*)^2^ + 0.3581*P*] where *P* = (*F*_o_^2^ + 2*F*_c_^2^)/3
4183 reflections	(Δ/σ)_max_ = 0.001
268 parameters	Δρ_max_ = 0.27 e Å^−^^3^
0 restraints	Δρ_min_ = −0.34 e Å^−^^3^

### Special details

**Table:**

Geometry. All s.u.'s (except the s.u. in the dihedral angle between two l.s. planes) are estimated using the full covariance matrix. The cell s.u.'s are taken into account individually in the estimation of s.u.'s in distances, angles and torsion angles; correlations between s.u.'s in cell parameters are only used when they are defined by crystal symmetry. An approximate (isotropic) treatment of cell s.u.'s is used for estimating s.u.'s involving l.s. planes.
Refinement. Refinement of *F*^2^ against ALL reflections. The weighted *R*-factor wR and goodness of fit *S* are based on *F*^2^, conventional *R*-factors *R* are based on *F*, with *F* set to zero for negative *F*^2^. The threshold expression of *F*^2^ > 2σ(*F*^2^) is used only for calculating *R*-factors(gt) etc. and is not relevant to the choice of reflections for refinement. *R*-factors based on *F*^2^ are statistically about twice as large as those based on *F*, and *R*- factors based on ALL data will be even larger.

### Fractional atomic coordinates and isotropic or equivalent isotropic displacement parameters (Å^2^)

**Table:**

	*x*	*y*	*z*	*U*_iso_*/*U*_eq_	
S1	0.51694 (5)	0.64771 (3)	0.10731 (6)	0.01767 (15)	
O1	0.86026 (16)	0.47833 (10)	1.1894 (2)	0.0440 (5)	
O2	0.66466 (16)	0.47631 (9)	1.16581 (19)	0.0344 (4)	
O3	1.09960 (18)	0.66615 (11)	−0.6358 (3)	0.0553 (6)	
O4	0.91528 (19)	0.66409 (11)	−0.7736 (2)	0.0519 (6)	
N1	0.50139 (16)	0.59578 (9)	0.4425 (2)	0.0182 (4)	
N2	0.50940 (17)	0.56838 (9)	0.5969 (2)	0.0191 (4)	
H2N	0.446 (2)	0.5655 (12)	0.649 (3)	0.029*	
N3	0.75746 (18)	0.48377 (10)	1.1061 (2)	0.0267 (5)	
N4	0.60955 (16)	0.69295 (9)	−0.1986 (2)	0.0181 (4)	
N5	0.65934 (16)	0.70405 (9)	−0.3383 (2)	0.0184 (4)	
H5N	0.611 (2)	0.7106 (11)	−0.442 (3)	0.028*	
N6	0.9889 (2)	0.66605 (11)	−0.6418 (3)	0.0340 (5)	
C1	0.38972 (19)	0.64722 (10)	0.2032 (2)	0.0171 (4)	
C2	0.29149 (19)	0.67487 (10)	0.1014 (3)	0.0194 (5)	
H2	0.2137	0.6787	0.1322	0.023*	
C3	0.31778 (19)	0.69709 (10)	−0.0543 (2)	0.0186 (4)	
H3	0.2596	0.7175	−0.1389	0.022*	
C4	0.43575 (19)	0.68609 (10)	−0.0706 (2)	0.0173 (4)	
C5	0.3983 (2)	0.61919 (10)	0.3698 (2)	0.0189 (5)	
H5	0.3297	0.6181	0.4242	0.023*	
C6	0.6201 (2)	0.54271 (10)	0.6765 (3)	0.0184 (5)	
C7	0.6332 (2)	0.52533 (10)	0.8470 (3)	0.0189 (5)	
H7	0.5680	0.5303	0.9069	0.023*	
C8	0.7441 (2)	0.50061 (11)	0.9255 (2)	0.0208 (5)	
C9	0.8416 (2)	0.49104 (11)	0.8444 (3)	0.0245 (5)	
H9	0.9165	0.4740	0.9028	0.029*	
C10	0.8256 (2)	0.50743 (11)	0.6740 (3)	0.0240 (5)	
H10	0.8903	0.5008	0.6140	0.029*	
C11	0.7166 (2)	0.53338 (10)	0.5900 (3)	0.0216 (5)	
H11	0.7076	0.5448	0.4738	0.026*	
C12	0.49410 (19)	0.70092 (10)	−0.2128 (2)	0.0174 (4)	
H12	0.4474	0.7164	−0.3159	0.021*	
C13	0.77989 (19)	0.68714 (10)	−0.3355 (2)	0.0162 (4)	
C14	0.8226 (2)	0.68469 (10)	−0.4875 (3)	0.0193 (5)	
H14	0.7703	0.6936	−0.5921	0.023*	
C15	0.9433 (2)	0.66899 (11)	−0.4818 (3)	0.0223 (5)	
C16	1.0241 (2)	0.65534 (12)	−0.3337 (3)	0.0264 (5)	
H16	1.1069	0.6452	−0.3345	0.032*	
C17	0.9786 (2)	0.65709 (11)	−0.1840 (3)	0.0253 (5)	
H17	1.0311	0.6473	−0.0802	0.030*	
C18	0.8583 (2)	0.67280 (10)	−0.1833 (3)	0.0200 (5)	
H18	0.8289	0.6738	−0.0796	0.024*	

### Atomic displacement parameters (Å^2^)

**Table:**

	*U*^11^	*U*^22^	*U*^33^	*U*^12^	*U*^13^	*U*^23^
S1	0.0173 (3)	0.0206 (3)	0.0161 (3)	0.0015 (2)	0.00585 (19)	0.0015 (2)
O1	0.0278 (11)	0.0719 (15)	0.0297 (10)	0.0132 (10)	−0.0019 (8)	0.0147 (9)
O2	0.0306 (10)	0.0526 (12)	0.0217 (8)	0.0002 (8)	0.0090 (7)	0.0075 (7)
O3	0.0333 (12)	0.0913 (18)	0.0497 (12)	0.0191 (11)	0.0300 (10)	0.0196 (11)
O4	0.0481 (13)	0.0905 (17)	0.0198 (10)	0.0207 (11)	0.0136 (9)	0.0044 (9)
N1	0.0241 (10)	0.0195 (10)	0.0122 (8)	−0.0030 (8)	0.0065 (7)	−0.0013 (7)
N2	0.0215 (10)	0.0240 (10)	0.0132 (8)	−0.0002 (8)	0.0069 (7)	0.0020 (7)
N3	0.0290 (12)	0.0286 (11)	0.0230 (10)	0.0034 (9)	0.0061 (9)	0.0030 (8)
N4	0.0212 (10)	0.0197 (10)	0.0152 (9)	−0.0012 (8)	0.0080 (7)	−0.0006 (7)
N5	0.0165 (9)	0.0272 (10)	0.0126 (9)	0.0029 (8)	0.0052 (7)	0.0040 (7)
N6	0.0326 (13)	0.0449 (14)	0.0297 (12)	0.0129 (10)	0.0192 (10)	0.0116 (9)
C1	0.0188 (11)	0.0167 (11)	0.0171 (10)	−0.0050 (9)	0.0070 (8)	−0.0024 (8)
C2	0.0161 (11)	0.0241 (12)	0.0186 (11)	−0.0013 (9)	0.0051 (8)	−0.0013 (8)
C3	0.0172 (11)	0.0227 (11)	0.0154 (10)	−0.0010 (9)	0.0020 (8)	0.0006 (8)
C4	0.0204 (11)	0.0164 (11)	0.0148 (10)	−0.0018 (9)	0.0025 (8)	−0.0001 (8)
C5	0.0208 (11)	0.0198 (11)	0.0174 (10)	−0.0039 (9)	0.0068 (8)	−0.0021 (8)
C6	0.0212 (12)	0.0151 (11)	0.0190 (10)	−0.0044 (9)	0.0042 (8)	−0.0020 (8)
C7	0.0219 (12)	0.0176 (11)	0.0181 (10)	−0.0018 (9)	0.0063 (8)	−0.0009 (8)
C8	0.0258 (13)	0.0207 (12)	0.0156 (10)	−0.0034 (9)	0.0032 (9)	0.0009 (8)
C9	0.0202 (12)	0.0260 (13)	0.0274 (12)	0.0003 (10)	0.0049 (9)	0.0012 (9)
C10	0.0211 (12)	0.0261 (13)	0.0274 (12)	−0.0029 (10)	0.0112 (9)	0.0001 (9)
C11	0.0245 (12)	0.0225 (12)	0.0186 (11)	−0.0053 (9)	0.0059 (9)	0.0009 (8)
C12	0.0190 (11)	0.0183 (11)	0.0150 (10)	0.0006 (9)	0.0033 (8)	−0.0009 (8)
C13	0.0173 (11)	0.0163 (11)	0.0162 (10)	−0.0012 (8)	0.0061 (8)	0.0001 (8)
C14	0.0200 (12)	0.0201 (11)	0.0186 (10)	0.0004 (9)	0.0060 (8)	0.0031 (8)
C15	0.0237 (12)	0.0255 (12)	0.0207 (11)	0.0030 (9)	0.0116 (9)	0.0019 (8)
C16	0.0149 (11)	0.0340 (14)	0.0313 (12)	0.0040 (10)	0.0070 (9)	0.0018 (10)
C17	0.0193 (12)	0.0349 (14)	0.0196 (11)	0.0007 (10)	−0.0016 (9)	−0.0011 (9)
C18	0.0234 (12)	0.0236 (12)	0.0137 (10)	−0.0002 (9)	0.0050 (8)	−0.0002 (8)

### Geometric parameters (Å, °)

**Table:**

S1—C1	1.737 (2)	C5—H5	0.9500
S1—C4	1.739 (2)	C6—C11	1.399 (3)
O1—N3	1.225 (3)	C6—C7	1.398 (3)
O2—N3	1.229 (2)	C7—C8	1.382 (3)
O3—N6	1.230 (3)	C7—H7	0.9500
O4—N6	1.217 (3)	C8—C9	1.382 (3)
N1—C5	1.288 (3)	C9—C10	1.390 (3)
N1—N2	1.352 (2)	C9—H9	0.9500
N2—C6	1.391 (3)	C10—C11	1.389 (3)
N2—H2N	0.89 (3)	C10—H10	0.9500
N3—C8	1.473 (3)	C11—H11	0.9500
N4—C12	1.285 (3)	C12—H12	0.9500
N4—N5	1.360 (2)	C13—C14	1.391 (3)
N5—C13	1.389 (3)	C13—C18	1.400 (3)
N5—H5N	0.92 (2)	C14—C15	1.380 (3)
N6—C15	1.468 (3)	C14—H14	0.9500
C1—C2	1.369 (3)	C15—C16	1.385 (3)
C1—C5	1.445 (3)	C16—C17	1.388 (3)
C2—C3	1.413 (3)	C16—H16	0.9500
C2—H2	0.9500	C17—C18	1.385 (3)
C3—C4	1.368 (3)	C17—H17	0.9500
C3—H3	0.9500	C18—H18	0.9500
C4—C12	1.449 (3)		
			
C1—S1—C4	91.17 (10)	C6—C7—H7	121.1
C5—N1—N2	118.56 (18)	C9—C8—C7	123.82 (19)
N1—N2—C6	119.09 (18)	C9—C8—N3	118.9 (2)
N1—N2—H2N	122.5 (16)	C7—C8—N3	117.29 (19)
C6—N2—H2N	118.4 (16)	C8—C9—C10	117.3 (2)
O1—N3—O2	123.37 (19)	C8—C9—H9	121.4
O1—N3—C8	118.43 (19)	C10—C9—H9	121.4
O2—N3—C8	118.20 (19)	C9—C10—C11	121.2 (2)
C12—N4—N5	117.41 (17)	C9—C10—H10	119.4
N4—N5—C13	119.06 (17)	C11—C10—H10	119.4
N4—N5—H5N	121.3 (16)	C10—C11—C6	119.9 (2)
C13—N5—H5N	117.1 (16)	C10—C11—H11	120.0
O4—N6—O3	123.4 (2)	C6—C11—H11	120.0
O4—N6—C15	118.4 (2)	N4—C12—C4	119.47 (18)
O3—N6—C15	118.2 (2)	N4—C12—H12	120.3
C2—C1—C5	129.17 (19)	C4—C12—H12	120.3
C2—C1—S1	111.39 (15)	N5—C13—C14	118.84 (18)
C5—C1—S1	119.43 (16)	N5—C13—C18	121.25 (18)
C1—C2—C3	113.04 (19)	C14—C13—C18	119.9 (2)
C1—C2—H2	123.5	C15—C14—C13	118.01 (19)
C3—C2—H2	123.5	C15—C14—H14	121.0
C4—C3—C2	112.95 (19)	C13—C14—H14	121.0
C4—C3—H3	123.5	C14—C15—C16	123.8 (2)
C2—C3—H3	123.5	C14—C15—N6	118.30 (19)
C3—C4—C12	128.36 (19)	C16—C15—N6	117.9 (2)
C3—C4—S1	111.45 (15)	C15—C16—C17	117.0 (2)
C12—C4—S1	120.17 (16)	C15—C16—H16	121.5
N1—C5—C1	118.30 (19)	C17—C16—H16	121.5
N1—C5—H5	120.9	C18—C17—C16	121.3 (2)
C1—C5—H5	120.9	C18—C17—H17	119.4
N2—C6—C11	121.80 (19)	C16—C17—H17	119.4
N2—C6—C7	118.27 (19)	C17—C18—C13	119.98 (19)
C11—C6—C7	119.9 (2)	C17—C18—H18	120.0
C8—C7—C6	117.9 (2)	C13—C18—H18	120.0
C8—C7—H7	121.1		
			
C5—N1—N2—C6	179.76 (19)	N3—C8—C9—C10	179.9 (2)
C12—N4—N5—C13	−170.79 (19)	C8—C9—C10—C11	1.1 (3)
C4—S1—C1—C2	0.37 (17)	C9—C10—C11—C6	−0.8 (3)
C4—S1—C1—C5	179.95 (17)	N2—C6—C11—C10	−179.8 (2)
C5—C1—C2—C3	−179.9 (2)	C7—C6—C11—C10	−0.6 (3)
S1—C1—C2—C3	−0.4 (2)	N5—N4—C12—C4	176.44 (18)
C1—C2—C3—C4	0.2 (3)	C3—C4—C12—N4	173.0 (2)
C2—C3—C4—C12	178.6 (2)	S1—C4—C12—N4	−8.6 (3)
C2—C3—C4—S1	0.1 (2)	N4—N5—C13—C14	165.61 (18)
C1—S1—C4—C3	−0.26 (17)	N4—N5—C13—C18	−14.5 (3)
C1—S1—C4—C12	−178.90 (17)	N5—C13—C14—C15	178.8 (2)
N2—N1—C5—C1	−178.75 (17)	C18—C13—C14—C15	−1.1 (3)
C2—C1—C5—N1	−180.0 (2)	C13—C14—C15—C16	0.2 (3)
S1—C1—C5—N1	0.5 (3)	C13—C14—C15—N6	179.7 (2)
N1—N2—C6—C11	−12.2 (3)	O4—N6—C15—C14	−15.1 (3)
N1—N2—C6—C7	168.57 (18)	O3—N6—C15—C14	164.4 (2)
N2—C6—C7—C8	−179.20 (19)	O4—N6—C15—C16	164.5 (2)
C11—C6—C7—C8	1.5 (3)	O3—N6—C15—C16	−16.0 (3)
C6—C7—C8—C9	−1.2 (3)	C14—C15—C16—C17	0.8 (4)
C6—C7—C8—N3	178.75 (18)	N6—C15—C16—C17	−178.7 (2)
O1—N3—C8—C9	19.0 (3)	C15—C16—C17—C18	−1.0 (4)
O2—N3—C8—C9	−161.5 (2)	C16—C17—C18—C13	0.1 (3)
O1—N3—C8—C7	−161.0 (2)	N5—C13—C18—C17	−178.9 (2)
O2—N3—C8—C7	18.6 (3)	C14—C13—C18—C17	0.9 (3)
C7—C8—C9—C10	−0.1 (3)		

### Hydrogen-bond geometry (Å, °)

**Table:**

Cg1 is the centroid of the S1,C1–C4 ring.

**Table:**

*D*—H···*A*	*D*—H	H···*A*	*D*···*A*	*D*—H···*A*
N2—H2n···O2^i^	0.89 (2)	2.27 (2)	3.103 (2)	156 (2)
C2—H2···O3^ii^	0.95	2.46	3.278 (3)	145
C18—H18···O4^iii^	0.95	2.48	3.241 (3)	137
C12—H12···Cg1^iv^	0.95	2.58	3.323 (2)	135

Symmetry codes: (i) −*x*+1, −*y*+1, −*z*+2; (ii) *x*−1, *y*, *z*+1; (iii) *x*, *y*, *z*+1; (iv) *x*, −*y*+3/2, *z*−1/2.
